# A modified Delphi survey to build expert consensus on the structure and content of an enhanced care pathway for cognitive changes after stroke in the UK

**DOI:** 10.1186/s12913-024-11551-6

**Published:** 2024-10-01

**Authors:** Georgina Hobden, Eugene Yee Hing Tang, Nele Demeyere

**Affiliations:** 1 Department of Experimental Psychology, University of Oxford, Oxford, UK; 2 Population Health Sciences Institute, Newcastle University, Newcastle, UK; 3 Nuffield Department of Clinical Neurosciences, University of Oxford, Oxford, UK

**Keywords:** Stroke, Cognition, United Kingdom, Rehabilitation, Recommendations, Care, Consensus

## Abstract

**Background:**

Enhancing long-term support for post-stroke cognitive impairment is a top research priority. Addressing current gaps in UK post-stroke cognitive care provision requires a pragmatic and scalable intervention that can be integrated within the existing stroke care pathway. This study aimed to develop consensus on an initial set of core features for a UK-based monitoring and psychoeducational intervention for cognitive changes after stroke.

**Methods:**

An expert panel of UK healthcare professionals and researchers participated in an online modified Delphi survey. Candidate intervention features were identified from clinical guidelines, existing literature, research team/collaborator expertise, and PPI group lived experience. Survey participants indicated whether they agreed/disagreed/had no opinion about including each candidate feature in the intervention and free-text responses were invited. We analysed responses for consensus (≥ 75% agreement) using descriptive statistics, with items not reaching consensus carried into subsequent rounds. Template analysis was used to identify similarities/differences in viewpoints for items that did not reach consensus.

**Results:**

Three survey rounds were completed by 36, 29 and 26 participants, respectively. Participants agreed reviews should include a stroke-specific cognitive screen (97% agree) and assessment of other psychological changes (low mood, anxiety, fatigue: 94%, 90%, 89% agree, respectively). They agreed stroke survivors should be offered at least one review, regardless of their cognitive profile in hospital. They agreed on the importance of various cognition-focused psychoeducation topics, and formal (100% agree) and informal (79% agree) training for those conducting reviews. Consensus was not reached on the review mode (in person/remote options: 67% agree), offering reviews one-year post-discharge to patients without acute cognitive impairments (68% disagree), or including a dementia screen (63% disagree) and/or neuropsychological assessment battery (58% disagree). However, there were similarities in participant viewpoints. For example, participants emphasised the importance of onwards referral where clinically indicated.

**Conclusions:**

The UK-based post-stroke monitoring and psychoeducation intervention was originally conceptualised as a cognitive care pathway, but expert participants agreed on the importance of simultaneously addressing related psychological changes (e.g. low mood, anxiety). There was clear consensus on a minimum set of intervention features. Recommendations outlined here may usefully inform local service improvements.

**Supplementary Information:**

The online version contains supplementary material available at 10.1186/s12913-024-11551-6.

## Introduction

Cognitive impairment affects 48–98% of patients in the first weeks after stroke [1–6]. Although a proportion of patients recover from early impairments [7, 8], prevalence of cognitive impairment in the months and years after stroke remains high [6, 9, 10], with post-stroke cognitive impairment having a substantial negative impact on quality of life, activity, and participation [11, 12].

National clinical guidelines in the United Kingdom (UK) recommend that post-stroke cognitive screening should be conducted as soon as possible [13] and the UK Sentinel Stroke National Audit Programme (SSNAP) confirmed that cognitive screening was conducted before discharge in the vast majority of cases in 2022–2023 [14]. However, despite recommendations that “patients’ psychological needs should be considered throughout the rehabilitation process” (p.23) and that resources should be “in place to consider and support the psychological needs of stroke patients throughout their stroke care pathway” (p.36) [15], only 56% of UK stroke survivors needing support for psychological changes after discharge received any in 2022–2023 [14]. Furthermore, recent systematic reviews have concluded that managing post-stroke cognitive impairment is one of the most frequently reported unmet needs over the long-term [16–18].

The NHS Long Term Plan [19] and Demand Signalling Report [20] have called for improved long-term care after stroke in the UK, with psychological support being highlighted as an aspect of care requiring particular attention [20, 21]. However, supporting post-stroke cognitive impairment over the longer term is a substantial challenge, as previous clinical trials have found no strong evidence for interventions to improve post-stroke cognitive functioning directly (see Cochrane reviews: [22–25]). Whilst cognitive rehabilitation focusing on monitoring, psychoeducation, and signposting may be beneficial [26–28], there is a current lack of high-quality evidence evaluating the efficacy of such interventions after stroke [29]. Furthermore, previously developed interventions may not be easily implementable and scalable within the existing UK stroke care pathway (e.g., [26]), given the substantial clinical expertise and time required to administer them.

This study sits within an iterative multistage research programme guided by the Medical Research Council (MRC) framework for complex intervention research [30, 31]. The aim of the ongoing research programme is to develop and assess the feasibility of a pragmatic and scalable UK-based monitoring and psychoeducational intervention to address cognitive changes after stroke [32]. This modified Delphi survey was conducted to establish an initial set of key features to include within the intervention based on evidence and stakeholder perspectives, in line with MRC recommendations [30]. The intervention was initially conceptualised as comprising post-discharge reviews to monitor cognitive functioning over the medium-long term after stroke, and accompanying psychoeducation about any post-stroke cognitive changes identified. In this study, we aimed to develop expert consensus on the following more specific questions to clarify the intervention design:

- Who should receive cognitive reviews? Specifically, should cognitive reviews be offered to those with and without cognitive impairment detected in the acute/subacute post-stroke stages and should review timepoints differ depending on acute-subacute cognitive profiles?
- How should cognitive reviews be conducted? Given recent increases in remote healthcare provision [33–35] and the availability of cognitive screening tools validated for online use [e.g., 36], should in person and/or online cognitive reviews be offered?
- What should cognitive reviews include? A wide variety of tools are used to assess post-stroke cognition in clinical practice [37], with approaches ranging from brief screens to comprehensive neuropsychological assessment batteries [38]. Which approach(es) should be routinely incorporated within the intervention and should reviews assess psychological changes beyond but closely related to cognitive impairment (e.g., low mood, anxiety)?
- What training might be required to support healthcare professionals responsible for administering cognitive reviews? Healthcare professionals in community-based stroke teams have reported a lack of confidence when conducting post-stroke cognitive assessments and few opportunities for training [39]. What type of training would best support skill development and confidence building?

## Methods

### Participants

We recruited an expert panel of practice-based experts and research-based experts to participate in this online modified Delphi survey. Participants were included if they met the following criteria, which were carefully crafted to ensure participants had sufficient topic expertise: (i) self-reported professional interest in and/or experience with cognition after stroke; and (ii) either an (allied) healthcare professional with at least five yearsof experience working with stroke survivors in the UK, or a researcher with at least one published peer-reviewed stroke research article within the last ten years as first, second, or last author.

We aimed to include participants with heterogeneous characteristics (e.g., different occupations and geographic locations).

Although we did not define the precise composition or size of the panel a priori, we anticipated a large proportion of participants would occupy a professional role involving some aspect of cognitive care (i.e., occupational therapist, clinical psychologist/neuropsychologist), given the above eligibility criteria. However, we did not limit our sample to these professions to ensure views held by those specialising in other disciplines would be captured, given the likely possibility that perspectives on post-stroke cognitive care differ across professional disciplines. Nevertheless, participants were required to have at least some professional interest and/or expertise with cognition after stroke regardless of their clinical specialty, as per the Delphi method which aims to capture expert views.

Practice-based and research-based experts were recruited through a snow-balling approach after emailing initial professional contacts and publicising the study on social media (Twitter/X).

### Survey design

We identified a set of candidate intervention features by drawing on existing literature [26–29, 40–43], clinical guidelines and recommendations [13, 44], expertise of the core study team and collaborators, as well as lived experience of our Patient and Public Involvement (PPI) group. This ensured candidate intervention features were grounded in high-quality empirical evidence, robust psychological theory, and stakeholder perspectives, in line with MRC recommendations [30, 31]. Having identified candidate intervention features, the research team decided whether to include them in the survey by weighing them against the following quality criteria, which were adapted from a previous co-production study [45] to suit the specific purpose of this research: (1) Relevance to target outcome (i.e., pragmatic and scalable post-stroke cognitive care intervention); (2) Ease of implementation within the scope and scale of the broader research programme (i.e., developing and evaluating the feasibility of a post-stroke cognitive care intervention: 32); (c) Ease of implementation within current UK clinical practice.

Each candidate intervention feature that was retained after this process was converted to a statement to be included as a survey item in the first modified Delphi survey round. For example, the candidate feature “Stroke-specific cognitive screen” was converted to the statement “Cognitive reviews should include a stroke-specific cognitive screen (15–20 minutes) (e.g., Oxford Cognitive Screen; OCS)”. Survey participants were asked to indicate whether they agreed, disagreed, or had no opinion about each statement. Categorical response options (i.e., agree, disagree, no opinion) were used to reduce participant burden, to make sure participants were clear about the consequences of their votes, and to ensure the final results were actionable in terms of developing the intervention. Participants were encouraged to provide further free-text comments in response to each statement and at the end of each survey round.

The survey was designed a priori to include two to four rounds, with the maximum set to account for likely participant attrition and fatigue [46]. The survey was designed to end when all items reached consensus, four rounds had been completed, or there was little indication that consensus would be reached on the outstanding statements (a priori definition: <5% movement towards consensus from previous round for all statements that had not yet reached consensus). We aimed to achieve a 100% response rate for each survey round but accepted a round as valid if the response rate was at least 70%, recognising likely participant attrition [46].

### Survey procedure

Potential participants were provided with a detailed information sheet that outlined the aim of the study and what would be involved. They were invited to follow a hyperlink to provide informed consent to participate and complete an online eligibility screening form (Microsoft Forms). Those who met the above eligibility criteria were contacted by email and invited to complete the first online survey round (Microsoft Forms) by following a hyperlink. In each survey round, before any survey questions were presented, participants were reminded of the study aims and encouraged to contact the research team by email if they had any questions. Participants were given ten working days to complete each survey round. A reminder was sent to participants to complete each round five working days after its opening. Participants who had not completed the round when it closed were excluded from subsequent round(s).

Once each round was closed, the research team analysed results, developed the subsequent survey round by identifying and carrying forwards any items that had not reached consensus, and created personalised summary sheets of the results from the previous survey round (Supplementary Materials). Personalised summary sheets presented participants with each statement that had not reached consensus, their own response to the statement, the overall group response and all anonymised free-text comments provided in response to the statement [46]. They were distributed to participants individually by email. As per the standard modified Delphi approach, participants were encouraged to reflect on and critically examine their own response in light of the overall group response and qualitative comments from other participants in order to promote consensus-building.

### Data analysis

R-Studio version 4.3.1 was used to calculate descriptive statistics from quantitative survey data. For each survey item, we calculated the number and percentage of participants who agreed/disagreed/had no opinion on including the candidate intervention feature within the cognitive care intervention. Consensus was defined a priori as ≥ 75% agree/disagree after excluding responses of ‘no opinion’ [47].

Free-text responses to the first and final survey rounds were analysed using template analysis [48, 49]. Free-text responses from the first survey round were coded inductively at a semantic level with the goal of identifying additional candidate intervention features that had not been identified by the research team when developing the survey. Free-text responses from the final round were also analysed inductively, with the goal of identifying any similarities and/or differences in participant viewpoints for statements that did not reach consensus by the end of the survey. Qualitative analyses were facilitated by Microsoft Word and Microsoft Excel.

While template analysis is a flexible qualitative analytic approach devoid of any specific epistemological or ontological framework [49], this study was positioned within a critical realist framework, where we acknowledged the active role of research team members in coding and interpreting the data, but conceptualised this interpretive role an asset, rather than a confounder. The research team remained mindful during the qualitative analytic process of their own professional disciplines, values, and existing beliefs about the potential cognitive care intervention.

## Results

**Fig. 1:**
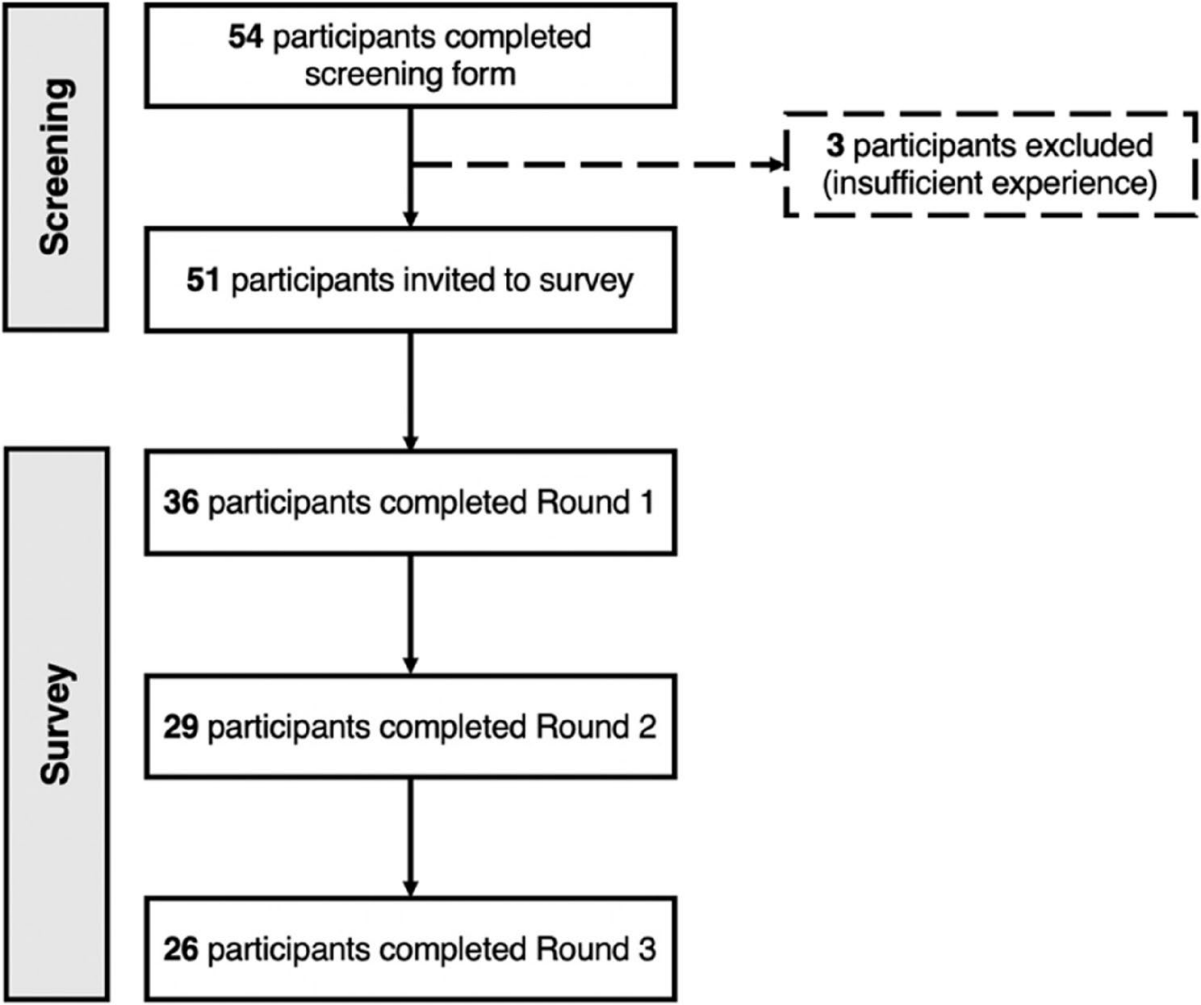
Flow diagram showing the number of participants included at each stage of the research

Figure 1 shows the number of participants who completed each stage of the research. Table 1 presents participant demographic details. Table 2 presents statements that reached consensus in each survey round and Table 3. presents statements that had not reached consensus by the end of the survey. The following sections summarise quantitative results from each survey round in turn. Results from the qualitative analysis conducted to identify overlapping viewpoints for those items that did not reach consensus by the end of the survey are presented briefly.

**Table 1:** Demographic details of the participants included in each survey round

Demographics, *N (%)*	Round 1 *N* = 36	Round 2 *N* = 29	Round 3 *N* = 26
Gender			
Male	7 (19.44)	5 (17.24)	5 (19.23)
Female	29 (80.56)	24 (82.76)	21 (80.77)
Years of age			
25-34	4 (11.11)	3 (10.34)	3 (11.54)
35-44	21 (58.33)	17 (58.62)	15 (57.69)
45-54	10 (27.78)	8 (27.59)	7 (26.92)
55-64	1 (2.78)	1 (3.45)	1 (3.85)
Professional occupation^a^			
Occupational therapist	15 (41.67)	12 (41.38)	11 (42.31)
Clinical psychologist	6 (16.67)	5 (17.24)	4 (15.38)
Clinical neuropsychologist	3 (8.33)	2 (6.90)	2 (7.69)
Speech and language therapist	2 (5.56)	2 (6.90)	2 (7.69)
Stroke specialist nurse	2 (5.56)	0 (0.00)	0 (0.00)
Physician	4 (11.11)	4 (13.79)	4 (15.38)
Professor or associate professor	1 (2.78)	1 (3.45)	1 (3.85)
Senior lecturer or lecturer	4 (11.11)	4 (13.79)	4 (15.38)
Postdoctoral researcher	3 (8.33)	3 (10.34)	2 (7.69)
Doctoral student	2 (5.56)	2 (6.90)	2 (7.69)
Region			
North East England	2 (5.56)	2 (6.90)	2 (7.69)
North West England	6 (16.67)	5 (17.24)	4 (15.38)
Yorkshire and the Humber	3 (8.33)	2 (6.90)	2 (7.69)
West Midlands	6 (16.67)	4 (13.79)	3 (11.54)
East of England	2 (5.56)	2 (6.90)	2 (7.69)
London	4 (11.11)	3 (10.34)	3 (11.54)
South East England	7 (19.44)	6 (20.69)	5 (19.23)
South West England	2 (5.56)	2 (6.90)	2 (7.69)
Scotland	4 (11.11)	3 (10.34)	3 (11.54)
Years of experience			
0-5	3 (8.33)	3 (10.34)	3 (11.54)
6-10	9 (25.00)	6 (20.69)	6 (23.08)
>10	24 (66.67)	20 (68.97)	17 (65.38)

^a^Participants were given the option to select more than one professional occupation, as several participants had both clinical and academic roles

**Table 2:** Items that reached consensus within the modified Delphi survey

Statement	Consensus	Round consensus reached	Respondents (*N*)		Agree *N* (%)	Disagree *N* (%)
			Total	Excluding no opinion		
Stroke survivors **with a cognitive impairment** detected during acute hospital admission should have a **review** of their cognition **after discharge** from an acute inpatient setting.	Agree	1	36	36	36 (100.00)	0 (0.00)
Stroke survivors **with no cognitive impairment** detected during acute hospital admission should have a **review** of their cognition **after discharge** from an acute inpatient setting.	Agree	1	36	34	30 (88.24)	4 (11.76)
For stroke survivors **with a cognitive impairment** detected during acute hospital admission, a review of their cognition should take place in the **first few weeks** after discharge.	Agree	1	36	35	30 (85.71)	5 (14.29)
For stroke survivors **with a cognitive impairment** detected during acute hospital admission, a review of their cognition should take place **3-months** after discharge.	Agree	1	31	27	25 (92.59)	2 (7.41)
For stroke survivors **with a cognitive impairment** detected during acute hospital admission, a review of their cognition should take place **6-months** after discharge.	Agree	1	30	22	21 (95.45)	1 (4.55)
For stroke survivors **with a cognitive impairment** detected during acute hospital admission, a review of their cognition should take place **1-year** after discharge.	Agree	1	29	20	19 (95.00)	1 (5.00)
For stroke survivors **with no cognitive impairment** detected during acute hospital admission, a review of their cognition should take place in the **first few weeks** after discharge.	Agree	1	32	28	23 (82.14)	5 (17.86)
For stroke survivors **with no cognitive impairment** detected during acute hospital admission, a review of their cognition should take place **3-months after discharge**.	Agree	2	29	28	23 (82.14)	5 (17.86)
For stroke survivors **with no cognitive impairment** detected during acute hospital admission, a review of their cognition should take place **6-months** after discharge.	Agree	1	29	20	16 (80.00)	4 (20.00)
Cognitive reviews should take place **in person**.	Agree	1	36	30	26 (86.67)	4 (13.33)
Cognitive reviews should include a **stroke-specific cognitive screen** (15–20 min) (e.g., Oxford Cognitive Screen; OCS).	Agree	1	36	33	32 (96.97)	1 (3.03)
Cognitive reviews should include a **questionnaire** for the **stroke survivor** about their post-stroke cognition.	Agree	1	36	34	33 (97.06)	1 (2.94)
Cognitive reviews should include a **questionnaire** for a **family member** about the stroke survivor’s cognition.	Agree	1	36	29	28 (96.55)	1 (3.45)
Cognitive reviews should include a **depression screen** (e.g., Patient Health Questionnaire-9; PHQ-9).	Agree	1	36	32	30 (93.75)	2 (6.25)
Cognitive reviews should include an **anxiety screen** (e.g., Generalised Anxiety Disorder Assessment-7; GAD-7).	Agree	1	36	31	28 (90.32)	3 (8.33)
Cognitive reviews should include a **general discussion** with the stroke survivor about their **overall cognitive functioning.**	Agree	1	36	34	34 (100.00)	0 (0.00)
Cognitive reviews should include a **general discussion** with the stroke survivor about their **domain-specific cognitive functioning.**	Agree	1	36	28	27 (96.43)	1 (3.57)
Stroke survivors should be told the **results of the cognitive assessment** conducted during the review.	Agree	1	36	32	30 (93.75)	2 (6.25)
Stroke survivors should be told how their **cognitive assessment result compares to earlier cognitive assessment results** (e.g., in cognitive screen completed in acute inpatient setting).	Agree	1	35	31	28 (90.32)	3 (9.68)
Stroke survivors should be told about **potential cognitive trajectories** during the review.	Agree	1	36	33	26 (78.79)	7 (21.21)
Stroke survivors should be told about the potential **impact** of any cognitive impairments on **activities of daily living** during the review.	Agree	1	36	34	34 (100.00)	0 (0.00)
Stroke survivors should be **signposted** to available **support and services** during the review.	Agree	1	36	36	36 (100.00)	0 (0.00)
Healthcare professionals responsible for conducting cognitive reviews after stroke should receive **formal training** (e.g., training videos) on **administering the cognitive assessment**.	Agree	1	36	35	35 (100.00)	0 (0.00)
Healthcare professionals responsible for conducting cognitive reviews after stroke should receive **informal training** (e.g., training from colleague) on **administering the cognitive assessment**.	Agree	1	35	28	22 (78.57)	6 (21.43)
Healthcare professionals responsible for conducting cognitive reviews after stroke should receive **formal training** (e.g., training videos) on **discussing cognition** with stroke survivors and family members.	Agree	1	36	34	33 (97.06)	1 (2.94)
Healthcare professionals responsible for conducting cognitive reviews after stroke should receive **informal training** (e.g., training from colleague) on **discussing cognition** with stroke survivors and family members.	Agree	1	35	30	26 (86.67)	4 (13.33)
^a ^Cognitive reviews should include a **fatigue measure** (e.g. Fatigue Severity Scale; FSS).	Agree	2	29	28	25 (89.29)	3 (10.71)
^**a**^**Online training** (e.g., training videos) should be offered to healthcare professionals administering cognitive reviews.	Agree	2	29	27	26 (96.30)	1 (3.70)
^**a**^**In person training** (e.g., training course) should be offered to healthcare professionals administering cognitive reviews.	Agree	2	29	25	21 (84.00)	4 (16.00)

^a^Item introduced in second round of the modified Delphi survey

### Quantitative results

#### Screening and consent

The screening form and consent form were completed by 54 participants, 51 (94.44%) of whom met the inclusion and exclusion criteria. The 3 participants who did not meet the criteria were healthcare professionals, but they did not have at least five years of experience working with stroke survivors in the UK.

#### Round 1

Of the 51 participants invited, 36 (70.59%) completed the first survey round, which included demographic questions and 30 survey items based on candidate intervention features. In summary, the expert panel agreed that stroke survivors with (100.00%) and without (88.24%) cognitive impairment detected during acute hospital admission should be offered at least one cognitive review. They agreed those with cognitive impairment should receive a review in the first weeks (85.71%), three months (92.59%), six-months (95.45%) and one-year (95.00%) after discharge. Whilst they agreed those without cognitive impairment detected should receive a review in the first weeks (82.14%) and six-months (80.00%) after discharge, participants did not reach consensus on whether they should receive reviews at three-months (72.00% agree) and one-year (55.00% agree) after discharge.

Whilst the expert panel agreed that cognitive reviews should be conducted in person (86.67%), they failed to reach consensus on a separate item on whether stroke survivors should be given the choice of whether to attend a review in person or remotely (72.41% agree). Nevertheless, they agreed that a stroke specific cognitive screen (96.97%), a depression screen (93.75%), and an anxiety screen (90.32%) should be administered during reviews. However, they did not agree on whether the cognitive review should also include a dementia screen (e.g. MoCA) (56.52% agree) and/or a neuropsychological assessment (e.g. RBANS) (62.50% agree). Nevertheless, they did agree that self-report questionnaires about cognition for stroke survivors (97.06%) and family members (96.55%) should be included.

Participants agreed on the importance of information provision and psychoeducation during the cognitive reviews. Specifically, participants agreed that stroke survivors should be given information about their cognitive assessment results, (93.75%), how these results compare to earlier cognitive testing (90.32%), potential cognitive trajectories (78.79%), the impact of cognitive problems on ADLs (100.00%), and information about services and support available (100.00%).

Participants also agreed training for healthcare professionals administering cognitive reviews was important. They agreed healthcare professionals should be offered formal (100.00%) and informal (78.57%) training on administering cognitive assessments, as well as formal (97.06%) and informal (86.67%) training on discussing cognition with stroke survivors and their family members.

Analysis of free-text responses from the first survey round resulted in the addition of three further survey items to the second round (see Table 2 starred items). These represented three further candidate intervention features: fatigue measure, online training, in person training.

#### Round 2

The second survey round was completed by 29 (80.56%) of the 36 participants invited. The second survey round included 8 items, 4 (50%) of which reached consensus.

All three of the items developed from free-text responses to statements in the first survey round reached consensus: Participants agreed the reviews should include a fatigue measure (89.29%), and that training for healthcare professionals should be offered online (96.30%) and in person (84.00%).

However, only one of the five items carried over from the first round reached consensus in the second round. Participants agreed that stroke survivors with no cognitive impairment detected during acute hospital admission should receive a review of their cognition three-months after discharge (82.14%), but they did not reach consensus on whether these stroke survivors should receive a review one-year after discharge (74.07% disagree). Participants failed to reach consensus on whether stroke survivors should be given the option to complete reviews in person or remotely (62.07% agree) and whether the review should also include a dementia screen (65.38% disagree) and/or a neuropsychological assessment battery (58.33% disagree).

#### Round 3

The third round of the survey was completed by 26 (89.66%) of the 29 participants invited. It included four items, none of which reached consensus. Compared to results of the second round, only one item moved closer to consensus (In person and remote review options: Agree − 62.07–66.67%). The level of consensus for one item remained the same in Round 3 compared to Round 2 (Inclusion of neuropsychological assessment battery: Disagree − 58.33%). Two items moved further from consensus (Reviews one-year after discharge for those without cognitive impairment acutely: Disagree − 74.07–68.00%; Inclusion of dementia screen: Disagree − 65.38–62.50%). Table 3. presents a summary of results for these items from each survey round.

**Table 3:** Items that did not reach consensus in the modified Delphi survey

**Statement**	**Round**	**Responses** ***(N)***		**Agree** ***N (%)***	**Disagree** ***N (%)***
		**Total**	**Excluding no opinion**		
For stroke survivors **with no cognitive impairment** detected during acute hospital admission, a review of their cognition should take place **1-year** after discharge.	1	26	20	11 (55.00)	9 (45.00)
	2	29	27	7 (25.93)	20 (74.07)
	3	25	25	8 (32.00)	17 (68.00)
Individual stroke survivors should **choose** whether they would prefer cognitive reviews to take place either **in person or remotely** (i.e., telephone or videoconferencing).	1	36	29	21 (72.41)	8 (27.59)
	2	29	29	18 (62.07)	11 (37.93)
	3	26	24	16 (66.67)	8 (33.33)
Cognitive reviews should include a **dementia screen** (10-15 min) (e.g., Montreal Cognitive Assessment; MoCA).	1	36	23	13 (56.52)	10 (43.48)
	2	29	26	9 (34.62)	17 (65.38)
	3	26	24	9 (37.50)	15 (62.50)
Cognitive reviews should include a **neuropsychological assessment battery** (>30 min) (e.g., Repeatable Battery for the Assessment of Neuropsychological Status; RBANS).	1	35	24	15 (62.50)	9 (37.50)
	2	29	24	10 (41.67)	14 (58.33)
	3	26	24	10 (41.67)	14 (58.33)

### Qualitative results from final survey round

Despite failing to reach consensus on four items, qualitative analysis of free-text responses indicated that many participants shared similar viewpoints about potential risks, benefits, and caveats of including the above candidate intervention features in the intervention. Table 4 presents a summary of the final theme structure developed from free-text responses to items that did not reach consensus and example verbatim quotations. Further detail is provided in Supplementary Materials, where specific themes and subthemes are described in greater depth with additional example quotations.

**Table 4:** Summary of theme structure developed to identify potential reasons for the lack of consensus on four statements in the modified Delphi survey

Survey item		Theme	Example quotation
1	For stroke survivors with no cognitive impairment detected during acute hospital admission, a review of their cognition should take place 1-year after discharge.	Too late to be useful.	“Should be earlier, as by a year post someone may have lost a job or relationships due to lack of support for cognitive changes by that point.” (P28, Speech and Language Therapist)
		Concerns about feasibility.	*“Given the evidence of the high prevalence of dementia post stroke*,* I think this would be a good opportunity to follow up on this. However*,* the resources needed would make it impossible and I do feel resources could be better allocated on those who really need the cognitive rehabilitation.”* (P2, Occupational Therapist)
		It depends on earlier cognitive reviews.	*“I think if no cognitive deficits are indicated at 3 months there is no need to review again.”* (P4, Occupational Therapist)
2	Individual stroke survivors should **choose** whether they would prefer cognitive reviews to take place either **in person or remotely** (i.e., telephone or videoconferencing).	Downsides of remote assessments.	*“Building rapport is so much easier in person (assuming they’d not previously met the assessor) and this is important for the person to do their best during the assessment. I think it’s probably also more likely that higher level difficulties would be picked up if seen in person. Not saying that remote options don’t have a place*,* I just don’t think patient preference is the right way to decide.”* (P19, Doctoral Student)
		Importance of choice and person-centredness.	*“I do think choice and accessibility is important therefore both videoconferencing and face to face should be offered. Not telephone consult. However the pros and cons of each should be clearly explained to the patient to enable them making an informed choice. My personal preference would be face to face but remote is better than a DNA.”* (P29, Occupational Therapist)
3	Cognitive reviews should include a **dementia screen** (10–15 min) (e.g., Montreal Cognitive Assessment; MoCA).	Risk of misinterpretation in stroke populations.	*“Standard dementia screens can potentially be misleading because of all the confounds in a stroke population i.e. neglect*,* dysphasia*,* motor problems. Whatever training you put in place*,* there will still be some people who take the standard cut-off (e.g. 88/100 on ACE-III) and say*,* ‘the score is below the cut-off and this suggests the person has dementia’.”* (P1, Clinical Psychologist & Clinical Neuropsychologist)
		Preference for situation- and person-specific approach.	*“A dementia screen would be very useful in certain situations: evidence of longitudinal cognitive decline either before stroke or months/years after the stroke; or a cognitive profile that doesn’t match the stroke eg dense amnestic picture with a posterior stroke. Stroke and neurodegenerative disorders often co-exist particularly in the older patients. […] So Dementia screen is important in some patients but not routinely in ALL patients.”* (P7, Physician & Lecturer)
4	Cognitive reviews should include a **neuropsychological assessment battery** (> 30 min) (e.g., Repeatable Battery for the Assessment of Neuropsychological Status; RBANS).	Concerns about feasibility.	*“I don’t think there will ever be enough people in stroke services to administer something that requires a more sophisticated understanding of psychometric assessment to make this workable - a ‘review’ will end up being too long and unwieldy.”* (P1, Clinical Psychologist & Clinical Neuropsychologist)
		Importance of onwards referral.	*“A two tiered approach with initial screening/triage to select those needing more detailed assessment seems a better use of resource.”* (P33, Physician)

## Discussion

Expert healthcare professionals and stroke researchers who participated in this modified Delphi survey reached consensus on the majority of design decisions for a monitoring and psychoeducational intervention addressing cognitive changes to be implemented within the UK stroke care pathway. Participants agreed reviews should be offered to all stroke survivors, regardless of their acute/subacute cognitive profile, and that these reviews should take place in person. They also agreed the reviews should include a stroke-specific cognitive screen, screening for other psychological changes (low mood, anxiety, and fatigue), and specific cognition-related psychoeducation topics (global and domain-specific cognitive functioning, changes in cognition where this information is available from previous assessments, potential cognitive trajectories, impact of cognitive changes on activities of daily living, and signposting to available support and services). Finally, participants agreed that both formal and informal training should be offered to healthcare professionals administering post-stroke cognitive reviews, with training offered in person and remotely. By the end of the survey process, consensus had not been reached on whether reviews should also be offered remotely, whether stroke survivors without cognitive impairments detected in hospital should receive a review one-year post-discharge, and whether reviews should also include a dementia screen and/or neuropsychological assessment battery. Still, free-text responses highlighted a number of shared views, including the importance of onwards referral where clinically indicated. Stakeholder input from this study will be integrated with findings from related research [40, 42, 43, 50] to inform the final design of an intervention addressing the well-evidenced need for psychological support after stroke [16–18, 51].

Participants agreed at least one review encompassing screening for cognitive and other psychological changes should be offered to all stroke survivors. The importance of reviewing psychological changes is indeed emphasised in UK clinical guidelines for stroke care [13] but audit and commissioning data indicate insufficient provision of longer term post-stroke psychological support across the UK [14, 52]. Furthermore, and perhaps most concerningly, evidence suggests provision of psychological support in the months after stroke has been slightly declining over recent years [14, 53], in spite of widespread calls to prioritise post-stroke psychological care provision [20, 21, 54]. While interventions have been developed with the potential to address these gaps in post-stroke psychological care provision in the UK [26–28], they are generally limited by the substantial clinical expertise and time required to administer them. When identifying candidate intervention features to include in this modified Delphi survey, we held in mind the substantial constraints on community stroke services within the UK and expert stakeholder participants further reinforced the need to balance quality and efficiency of care provision. As a result, the monitoring and psychoeducational intervention that will be developed from this modified Delphi survey and related research may have the potential to fill key gaps in post-stroke care provision, without overburdening clinical services.

Participants also agreed the reviews should include a stroke-specific cognitive screen, a mood and anxiety screen, a fatigue measure, and psychoeducation about cognition. However, as the original focus of the intervention was on post-stroke cognitive care, participants were not asked whether psychoeducation about mood, anxiety, and fatigue should also be provided. While ongoing research is seeking to address this and other unresolved intervention design decisions through a rigorous iterative co-production process, a holistic treatment approach may be optimal, given the well-evidenced interconnectedness between post-stroke cognitive impairment and other psychological changes. The association between post-stroke cognitive impairment and low mood in particular has been robustly demonstrated, with previous studies reporting a significantly higher prevalence of post-stroke depression among stroke survivors with cognitive impairment compared to those without [55]. Furthermore, longitudinal studies have consistently indicated that cognitive impairment is a risk factor for developing post-stroke depression [56]. Though empirical evidence is weaker, symptoms of post-stroke depression may also have an indirect negative impact on post-stroke cognitive outcomes by reducing motivation to participate in rehabilitation and social activity, which are crucial for cognitive recovery after stroke [57]. Ultimately, given the robust and potentially bidirectional relationship between post-stroke cognitive impairment and low mod in particular, post-stroke cognitive care may ultimately prove more effective when other psychological changes are addressed in tandem.

Participants failed to reach consensus on four key design decisions, including whether reviews should additionally involve a dementia screen and/or neuropsychological assessment battery as a standard part of follow-up. This lack of consensus mirrors the ongoing debate within the literature and clinical practice about the optimal approach for post-stroke cognitive screening and assessment [38]. Both within this survey and in previous research, proponents of post-stroke dementia screening have emphasised the increased risk of developing dementia after stroke and thus the urgent need to screen for it [58]. Indeed, a recent meta-analysis reported that approximately 20% of stroke survivors experience clinically defined dementia one-year after stroke [59]. However, as acknowledged by participants in this study, results on dementia screens like the Montreal Cognitive Assessment (MoCA: [57]) and Mini-Mental State Examination (MMSE: [58]) may be confounded by stroke-specific deficits and lack sensitivity [60, 61], leading to potential misinterpretation and misdiagnoses when administered and interpreted by individuals with limited expertise. With regards to neuropsychology assessments, while proponents of neuropsychology assessments have emphasised the advantage of understanding cognitive profiles in detail to support rehabilitation, participants in this study highlighted the substantial constraints on clinical services in the UK and argued that it would not be feasible to implement extensive neuropsychological testing within a standardised and widespread post-stroke psychological care pathway. In both cases, participants emphasised the importance of onwards referral to specialist services, such as memory clinics and neuropsychology services.

In terms of limitations, the survey was designed and ethical approval was obtained prior to the publication of the latest revision of the National Clinical Guideline for Stroke in April 2023 [13], meaning that survey items were informed by the earlier 2016 guideline [62]. Nevertheless, our results echo recommendations from the updated clinical guideline, as well as providing important expansion on some aspects of psychological care provision (e.g., importance of providing specific information about assessment results, potential trajectories, impact of cognitive changes on activities of daily living, and signposting). A second limitation is that we only sought consensus on relatively broad intervention design decisions. For example, participants agreed that the intervention should include a stroke-specific cognitive screen, but we did not ask participants to advise on the optimal stroke-specific screening tool. We constrained the survey to broader design decisions to reduce participant burden and encourage participation. Finally, we note that a large proportion of participants were occupational therapists. While we do not consider this to be a study-specific limitation, but rather a consequence of recruiting participants with topic expertise as per the Delphi survey method, viewpoints on post-stroke cognitive care that are prevalent within other disciplines may be less well-reflected within the results of this study.

In conclusion, this study established expert consensus on an initial set of key intervention features for a Level 1 monitoring and psychoeducation intervention addressing post-stroke psychological changes to be integrated in the existing UK stroke care pathway. Additional research is currently underway to address design decisions that remain unresolved from this modified Delphi survey. Once the design of the intervention is finalised, future research will evaluate its feasibility and the extent to which the intervention has a clinically meaningful impact on patient outcomes. In the meantime, the recommendations outlined here may prove beneficial for informing local service improvements.

## Data Availability

No further data will be made available.

## Supplementary Infotmation

Supplementary Material 1.

## Data availability

No datasets were generated or analysed during the current study.
