# Supplementary material for: A modified Delphi survey to build expert consensus on the structure and content of an enhanced care pathway for cognitive changes after stroke in the UK
Source: BMC Health Serv Res. 2024 Oct 1;24:1162. doi: 10.1186/s12913-024-11551-6 (PMC11446070; doi:10.1186/s12913-024-11551-6)
Supplement: Supplementary file 1 — Supplementary Material 1. [file 12913_2024_11551_MOESM1_ESM.docx]

**Supplementary Materials**

**Supplementary Material.** Modified Delphi survey questions from each survey round.

***Round One***

*The cognitive care pathway will include****at least one post-stroke review of cognition****conducted by****Early Supported Discharge****stroke services in the UK.*

*We want to****establish consensus****on****who****should be offered these reviews,****when****reviews should take place,****where****they should be conducted,****what****they should include, and what****training****should be offered to healthcare professionals conducting the reviews.

Please respond to the* ***demographic*** *questions. Then, please read each statement and indicate whether you****agree****or****disagree****and provide any****further comments****you have about the statements.*

1. What is your **gender**?

Male

Female

Nonbinary

Prefer not to say

2. What is your current **age**?

18-24 years

25-34 years

35-44 years

45-54 years

55-64 years

65 years and over

Prefer not to say

3. What is your **professional** **occupation**? You may select any that apply.

Occupational Therapist

Clinical Psychologist

Clinical Neuropsychologist

Trainee Clinical Psychologist

Speech and Language Therapist

Stroke Specialist Nurse

Physiotherapist

Physician (e.g., Neurologist, Stroke Doctor, GP)

Professor or Associate Professor

Senior Lecturer or Lecturer

Clinical Research Fellow

Postdoctoral Researcher

Doctoral Student

Other _____

Prefer not to say

4. In which **region** of the **United Kingdom** are you based **professionally**?

North East England

North West England

Yorkshire and the Humber

East Midlands

West Midlands

East of England

London

South East England

South West England

Wales

Scotland

Northern Ireland

Prefer not to say

5. How many **years of experience** do you have working clinically with and/or researching **stroke**?

0-5 years

6-10 years

>10 years

Prefer not to say

6. Please select the **option** that best describes your **professional role** and **responsibilities**.

I **oversee** and/or **conduct** cognitive screening after stroke in clinical practice.

I have **professional expertise** in cognition after stroke but I am not responsible for overseeing and/or conducting cognitive screening after stroke in clinical practice.

Prefer not to say

7. Stroke survivors **with** **a cognitive impairment** detected during acute hospital admission should have a **review** of their cognition **after discharge**from an acute inpatient setting.

Agree

Disagree

No opinion

8. Please enter any **further comments** about the above statement.

_____________

9. Stroke survivors **with no cognitive impairment** detected during acute hospital admission should have a **review** of their cognition **after discharge**from an acute inpatient setting.

Agree

Disagree

No opinion

10. Please enter any **further comments** about the above statement.

_____________

11. For stroke survivors **with** **a cognitive impairment** detected during acute hospital admission, a review of their cognition should take place:

1. In the **first few weeks** after discharge.

Agree

Disagree

No opinion

1. **3-months** after discharge.

Agree

Disagree

No opinion

1. **6-months** after discharge.

Agree

Disagree

No opinion

1. 1**-year** after discharge.

Agree

Disagree

No opinion

12. Please enter any **further comments** about the above statements.

_____________

13. For stroke survivors **with no cognitive impairment** detected during acute hospital admission, a review of their cognition should take place:

1. In the **first few weeks** after discharge.

Agree

Disagree

No opinion

1. **3-months** after discharge.

Agree

Disagree

No opinion

1. **6-months** after discharge.

Agree

Disagree

No opinion

1. 1**-year** after discharge.

Agree

Disagree

No opinion

14. Please enter any **further comments** about the above statements.

_____________

15. Cognitive reviews should take place **in person**.

Agree

Disagree

No opinion

16. Please enter any **further comments** about the above statement.

_____________

17. Individual stroke survivors should **choose** whether they would prefer cognitive reviews to take place either **in person or remotely** (i.e., telephone or videoconferencing).

Agree

Disagree

No opinion

18. Please enter any **further comments** about the above statement.

_____________

19. Cognitive reviews should include a **dementia screen** (10-15 minutes) (e.g., Montreal Cognitive Assessment; MoCA).

Agree

Disagree

No opinion

20. Please enter any **further comments** about the above statement.

_____________

21. Cognitive reviews should include a **stroke-specific cognitive screen** (15-20 minutes) (e.g., Oxford Cognitive Screen; OCS).

Agree

Disagree

No opinion

22. Please enter any **further comments** about the above statement.

_____________

23. Cognitive reviews should include a **neuropsychological assessment battery** (>30 minutes) (e.g., Repeatable Battery for the Assessment of Neuropsychological Status; RBANS).

Agree

Disagree

No opinion

24. Please enter any **further comments** about the above statement.

_____________

25. Cognitive reviews should include a **questionnaire** for the **stroke survivor** about their post-stroke cognition.

Agree

Disagree

No opinion

26. Please enter any **further comments** about the above statement.

_____________

27. Cognitive reviews should include a **questionnaire** for a **family member** about the stroke survivor’s cognition.

Agree

Disagree

No opinion

28. Please enter any **further comments** about the above statement.

_____________

29. Cognitive reviews should include a **depression screen** (e.g., Patient Health Questionnaire-9; PHQ-9).

Agree

Disagree

No opinion

30. Please enter any **further comments** about the above statement.

_____________

31. Cognitive reviews should include an **anxiety screen** (e.g., Generalised Anxiety Disorder Assessment-7; GAD-7).

Agree

Disagree

No opinion

32. Please enter any **further comments** about the above statement.

_____________

33. Cognitive reviews should include a **general** **discussion** with the stroke survivor about their **overall cognitive functioning.**

Agree

Disagree

No opinion

34. Please enter any **further comments** about the above statement.

_____________

35. Cognitive reviews should include a **general** **discussion** with the stroke survivor about their **domain-specific cognitive functioning.**

Agree

Disagree

No opinion

36. Please enter any **further comments** about the above statement.

_____________

37. Stroke survivors should be told the **results of the cognitive assessment**conducted during the review.

Agree

Disagree

No opinion

38. Please enter any **further comments** about the above statement.

_____________

39. Stroke survivors should be told how their **cognitive assessment result compares to earlier cognitive assessment results**(e.g., in cognitive screen completed in acute inpatient setting).

Agree

Disagree

No opinion

40. Please enter any **further comments** about the above statement.

_____________

41. Stroke survivors should be told about **potential cognitive trajectories**during the review.

Agree

Disagree

No opinion

42. Please enter any **further comments** about the above statement.

_____________

43. Stroke survivors should be told about the potential **impact** of any cognitive impairments on **activities of daily living** during the review.

Agree

Disagree

No opinion

44. Please enter any **further comments** about the above statement.

_____________

45. Stroke survivors should be **signposted** to available **support and services**during the review.

Agree

Disagree

No opinion

46. Please enter any **further comments** about the above statement.

_____________

47. Healthcare professionals responsible for conducting cognitive reviews after stroke should receive **formal training** (e.g., training videos) on **administering the cognitive assessment**.

Agree

Disagree

No opinion

48. Please enter any **further comments** about the above statement.

_____________

49. Healthcare professionals responsible for conducting cognitive reviews after stroke should receive **informal training** (e.g., training from colleague) on **administering the cognitive assessment**.

Agree

Disagree

No opinion

50. Please enter any **further comments** about the above statement.

_____________

51. Healthcare professionals responsible for conducting cognitive reviews after stroke should receive **formal training** (e.g., training videos) on **discussing cognition**with stroke survivors and family members.

Agree

Disagree

No opinion

52. Please enter any **further comments** about the above statement.

_____________

53. Healthcare professionals responsible for conducting cognitive reviews after stroke should receive **informal training** (e.g., training from colleague) on **discussing cognition**with stroke survivors and family members.

Agree

Disagree

No opinion

54. Please enter any **further comments** about the above statement.

_____________

55. Please provide **any further thoughts** about follow-up reviews of cognition after stroke.

_____________

***Round Two***

*The cognitive care pathway will include****at least one post-stroke review of cognition****conducted by****Early Supported Discharge****stroke services in the UK.*

*We want to****establish consensus****on****who****should be offered these reviews,****when****reviews should take place,****where****they should be conducted,****what****they should include, and what****training****should be offered to healthcare professionals conducting the reviews.*

*Please read the* ***personalised feedback sheet*** *that has been emailed to you and reflect on the information provided before responding to this survey round.*

*Then, please read each statement below and indicate whether you****agree****or****disagree****and provide any****further comments****you have about the statements.*

1. For stroke survivors **with no cognitive impairment** detected during acute hospital admission, a review of their cognition should take place **3-months after discharge**.

Agree

Disagree

No opinion

2. Please enter any **further comments** about the above statement.

_____________

3. For stroke survivors **with no cognitive impairment** detected during acute hospital admission, a review of their cognition should take place **1-year after discharge**.

Agree

Disagree

No opinion

4. Please enter any **further comments** about the above statement.

_____________

5. Individual stroke survivors should **choose** whether they would prefer cognitive reviews to take place either **in person or remotely** (i.e., telephone or videoconferencing).

Agree

Disagree

No opinion

6. Please enter any **further comments** about the above statement.

_____________

7. Cognitive reviews should include a **dementia screen** (10-15 minutes) (e.g., Montreal Cognitive Assessment; MoCA).

Agree

Disagree

No opinion

8. Please enter any **further comments** about the above statement.

_____________

9. Cognitive reviews should include a **neuropsychological assessment battery** (>30 minutes) (e.g., Repeatable Battery for the Assessment of Neuropsychological Status; RBANS).

Agree

Disagree

No opinion

10. Please enter any **further comments** about the above statement.

_____________

11. Cognitive reviews should include a **fatigue measure** (e.g. Fatigue Severity Scale; FSS).

Agree

Disagree

No opinion

12. Please enter any **further comments** about the above statement.

_____________

13. **Online training**(e.g., training videos) should be offered to healthcare professionals administering cognitive reviews.

Agree

Disagree

No opinion

14. Please enter any **further comments** about the above statement.

_____________

15. **In person training**(e.g., training course) should be offered to healthcare professionals administering cognitive reviews.

Agree

Disagree

No opinion

16. Please enter any **further comments** about the above statement.

_____________

17. Please provide any **further thoughts** about follow-up reviews of cognition after stroke.

_____________

***Round Three***

*The cognitive care pathway will include****at least one post-stroke review of cognition****conducted by****Early Supported Discharge****stroke services in the UK.*

*We want to****establish consensus****on****who****should be offered these reviews,****when****reviews should take place,****where****they should be conducted,****what****they should include, and what****training****should be offered to healthcare professionals conducting the reviews.*

*Please read the* ***personalised feedback sheet*** *that has been emailed to you and reflect on the information provided before responding to this survey round.*

*Then, please read each statement below and indicate whether you****agree****or****disagree****and provide any****further comments****you have about the statements.*

1. For stroke survivors **with no cognitive impairment** detected during acute hospital admission, a review of their cognition should take place **1-year after discharge**.

Agree

Disagree

No opinion

2. Please enter any **further comments** about the above statement.

_____________

3. Individual stroke survivors should **choose** whether they would prefer cognitive reviews to take place either **in person or remotely** (i.e., telephone or videoconferencing).

Agree

Disagree

No opinion

4. Please enter any **further comments**about the above statement.

_____________

5. Cognitive reviews should include a **dementia screen** (10-15 minutes) (e.g., Montreal Cognitive Assessment; MoCA).

Agree

Disagree

No opinion

6. Please enter any **further comments** about the above statement.

_____________

7. Cognitive reviews should include a **neuropsychological assessment battery** (>30 minutes) (e.g., Repeatable Battery for the Assessment of Neuropsychological Status; RBANS).

Agree

Disagree

No opinion

8. Please enter any **further comments** about the above statement.

_____________

9. Please provide **any** **further thoughts** about follow-up reviews of cognition after stroke.

_____________

**Supplementary Figures.** Example participant feedback from the first round of the modified Delphi survey. Qualitative comments are presented verbatim with typographical errors uncorrected.

**
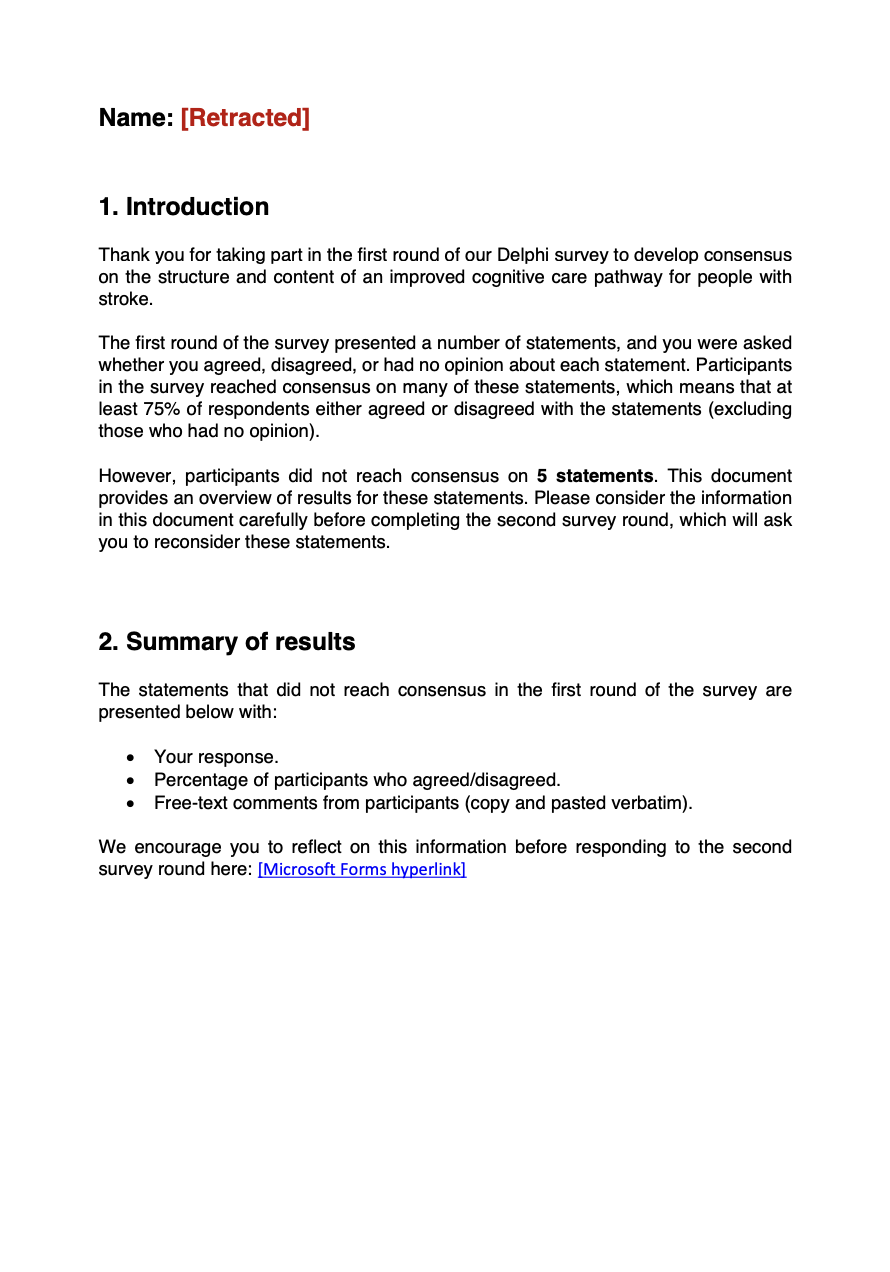
**

**
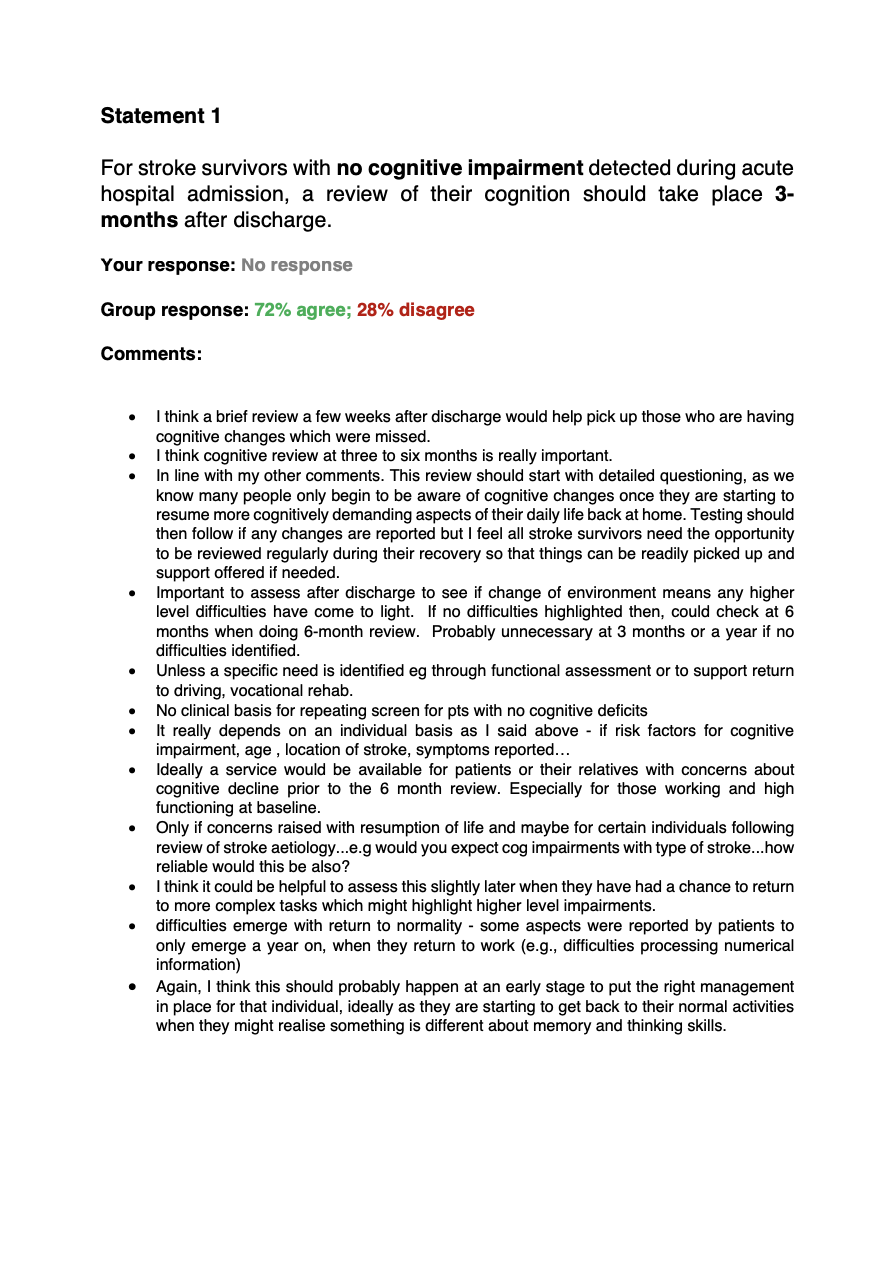
**

**
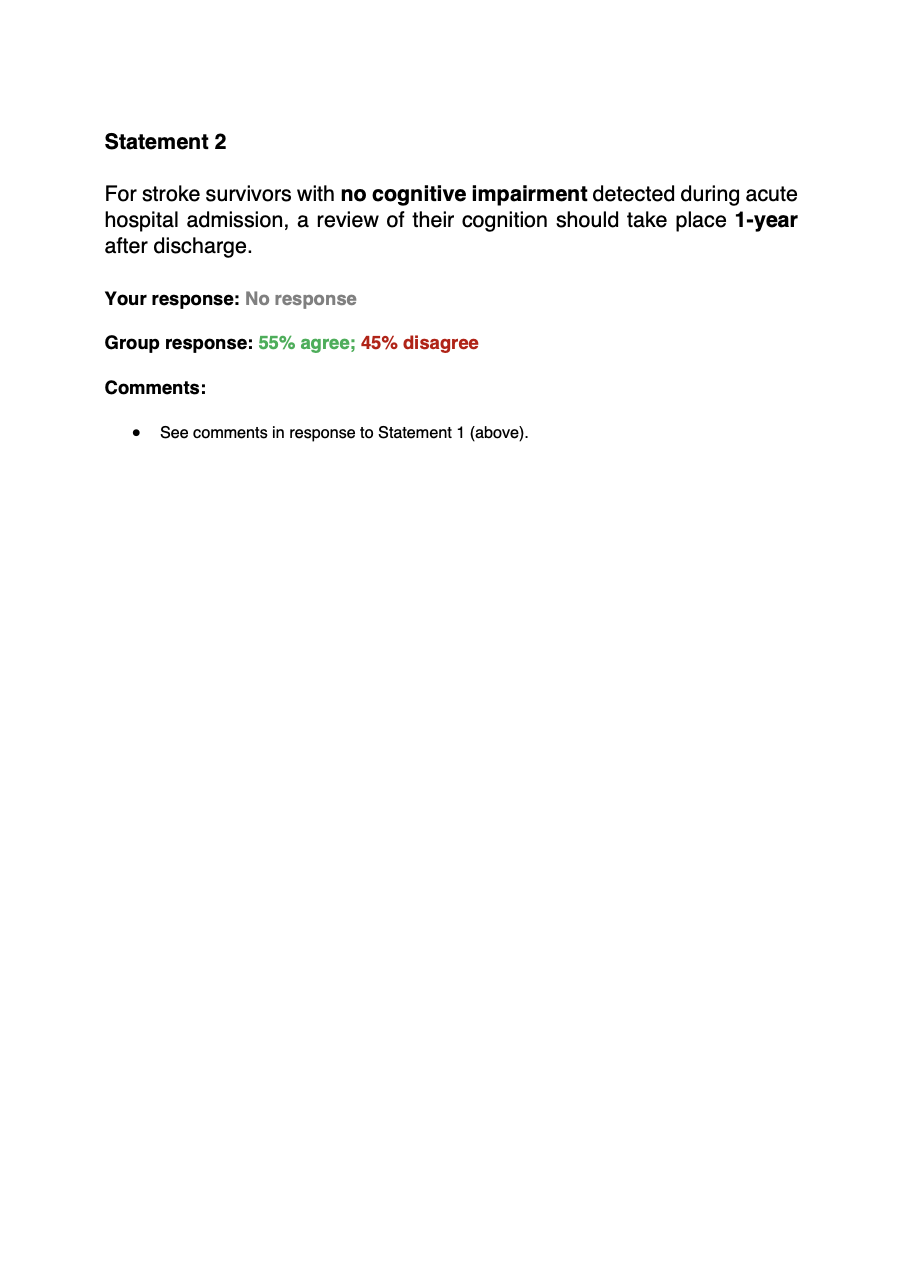
**

**
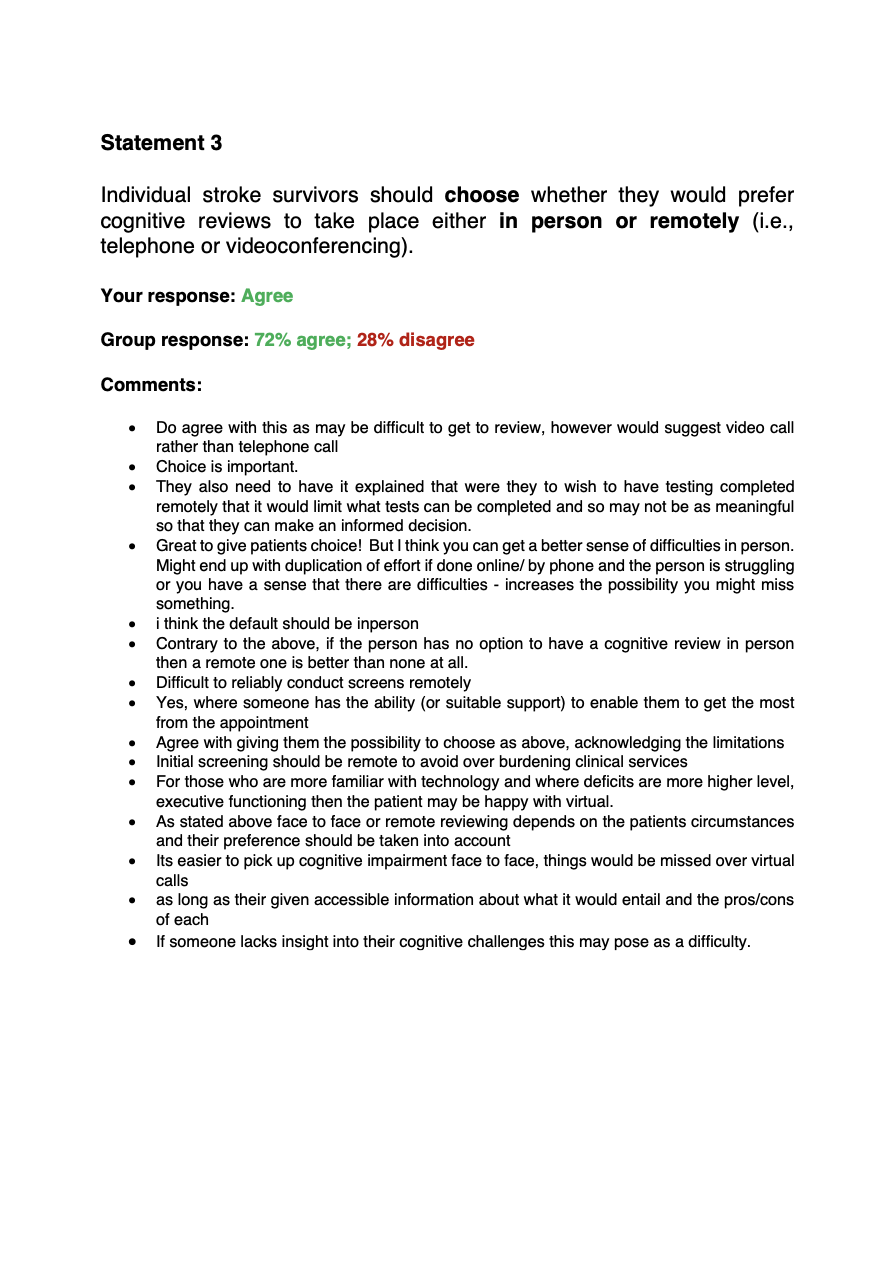
**

**
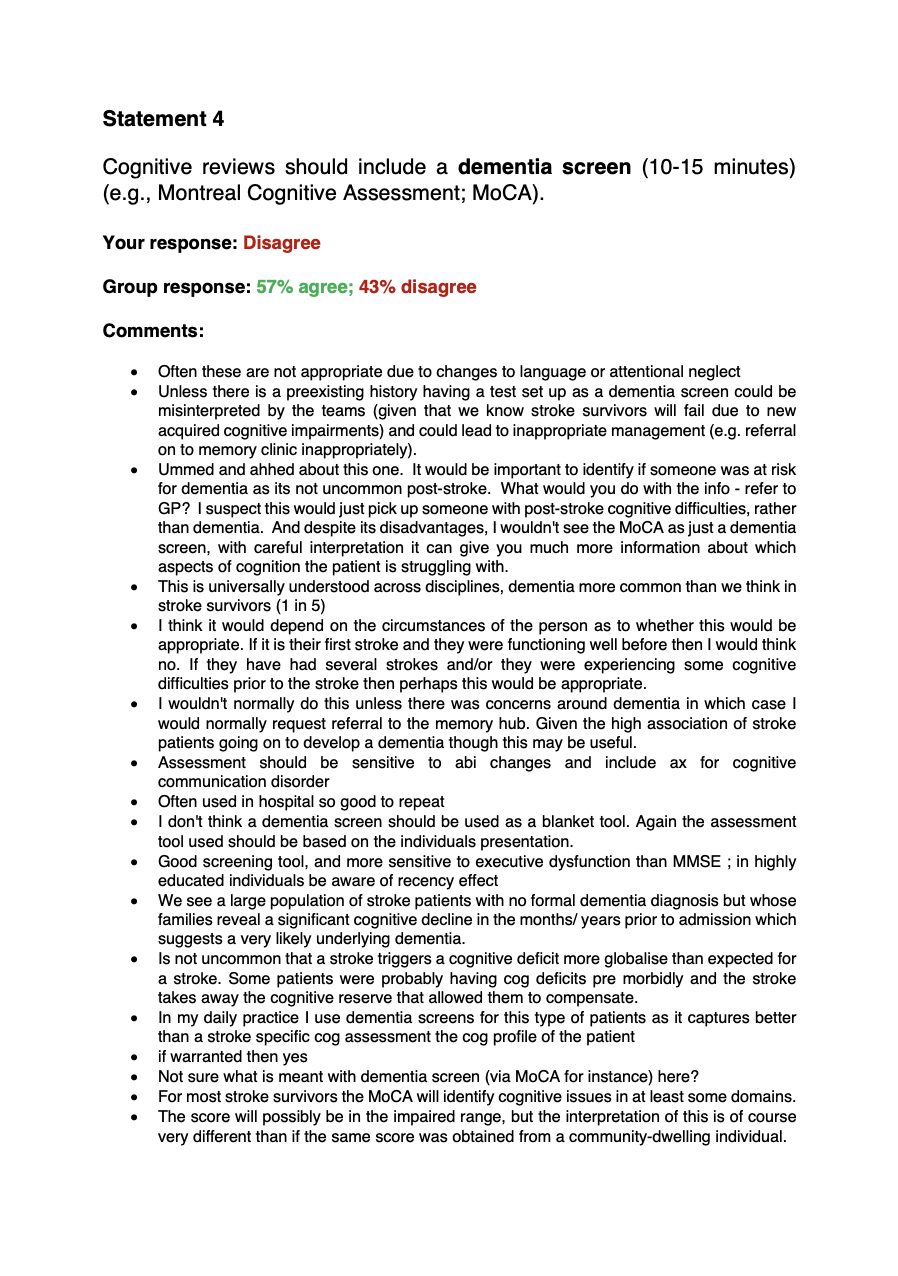
**

**
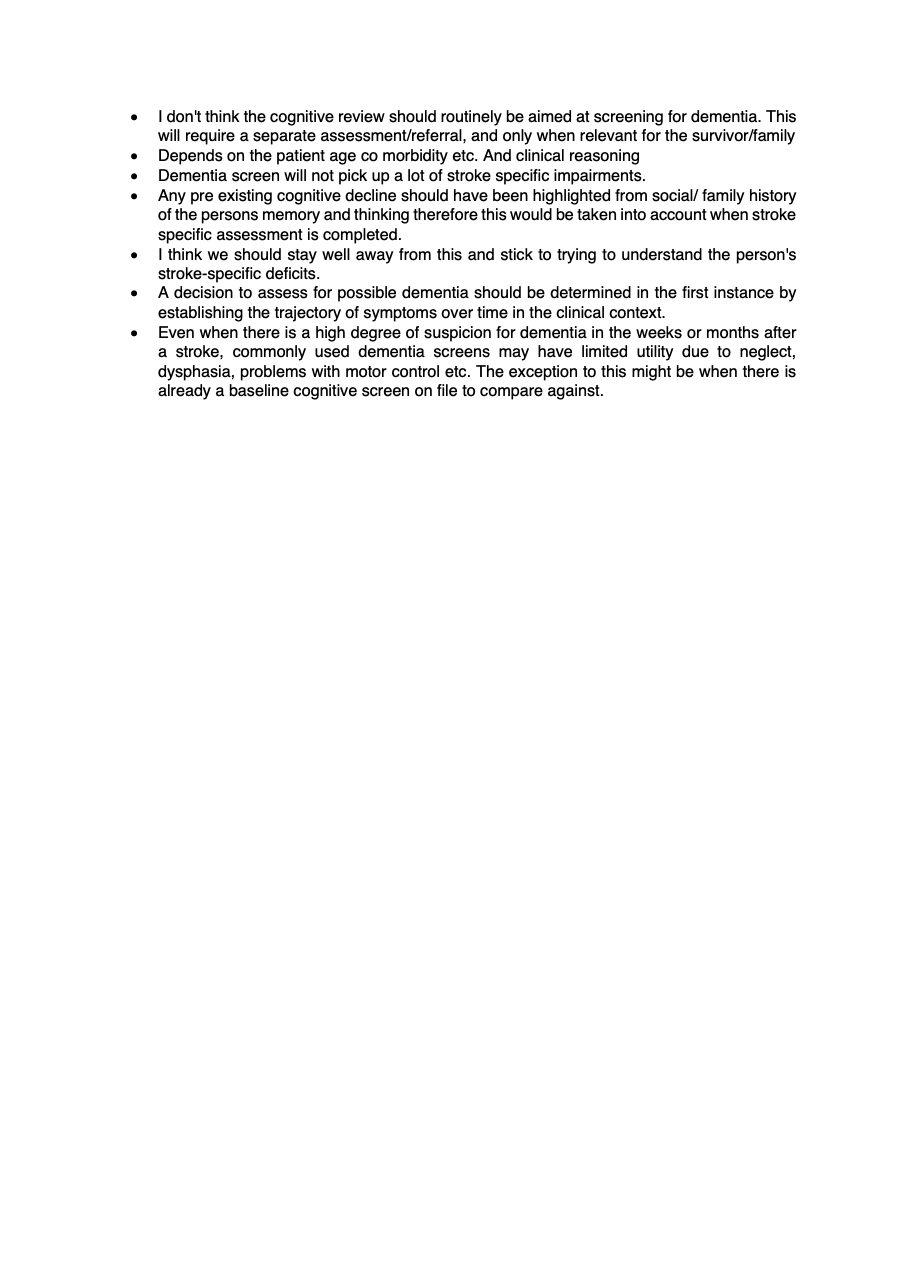
**

**
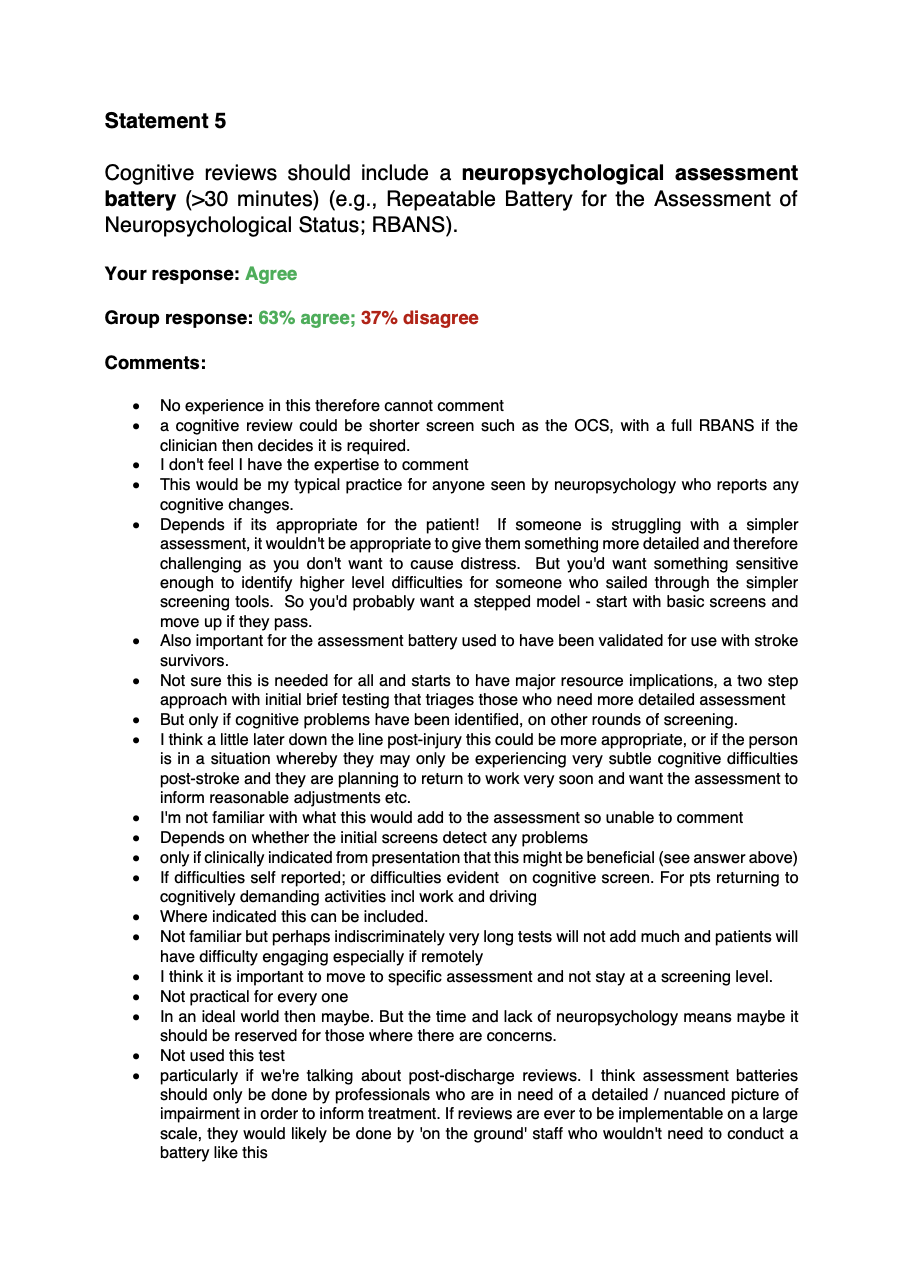
**

**
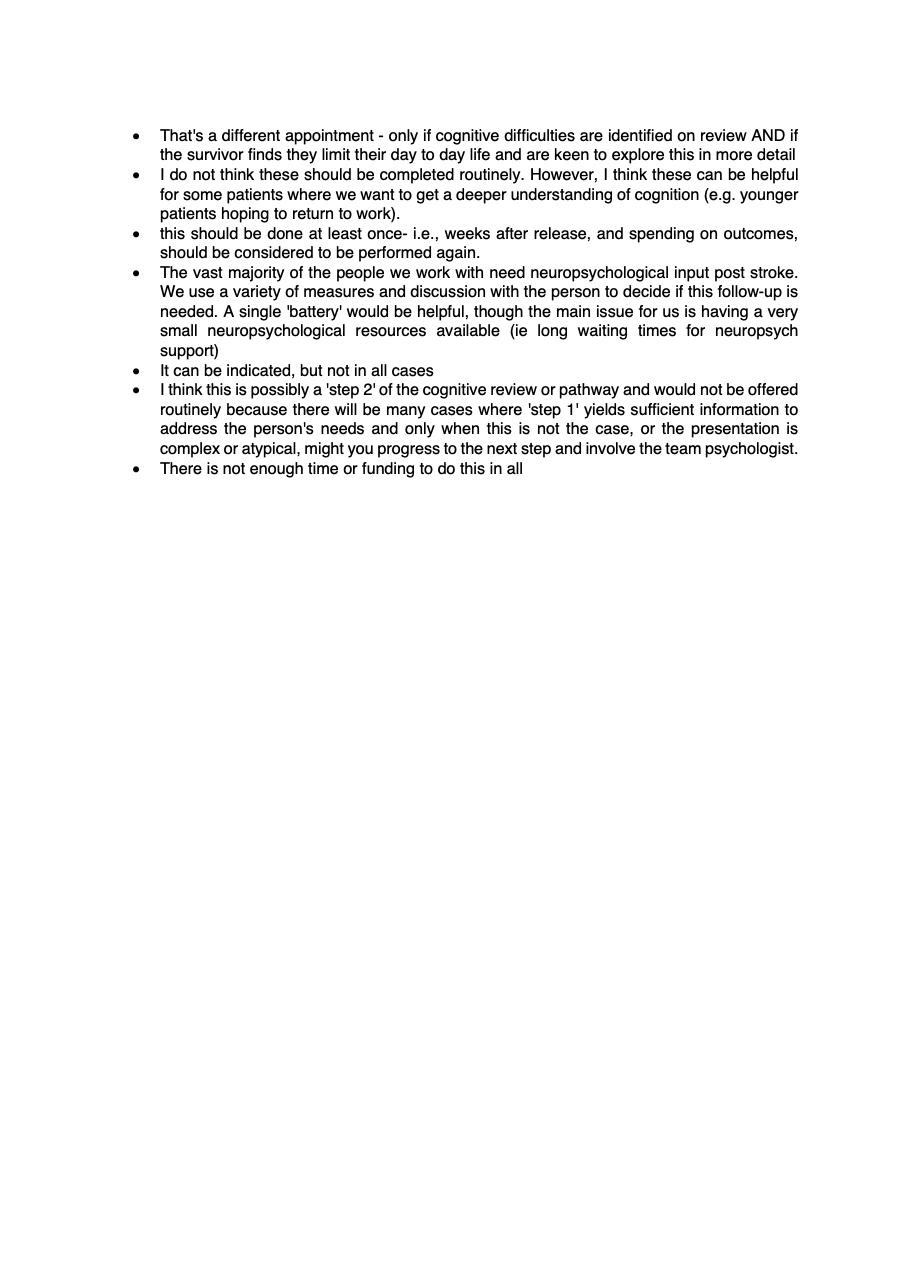
**

**
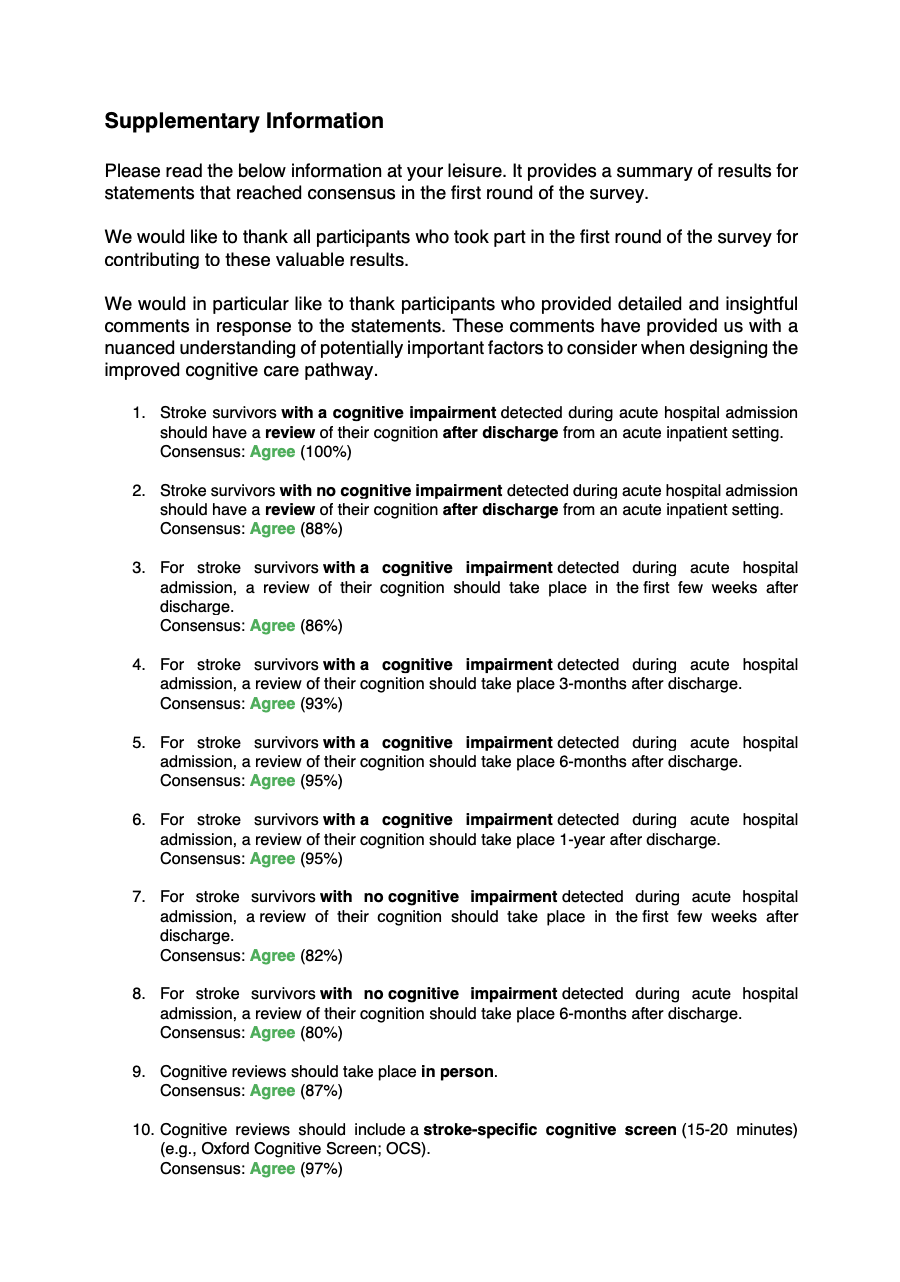
**

**
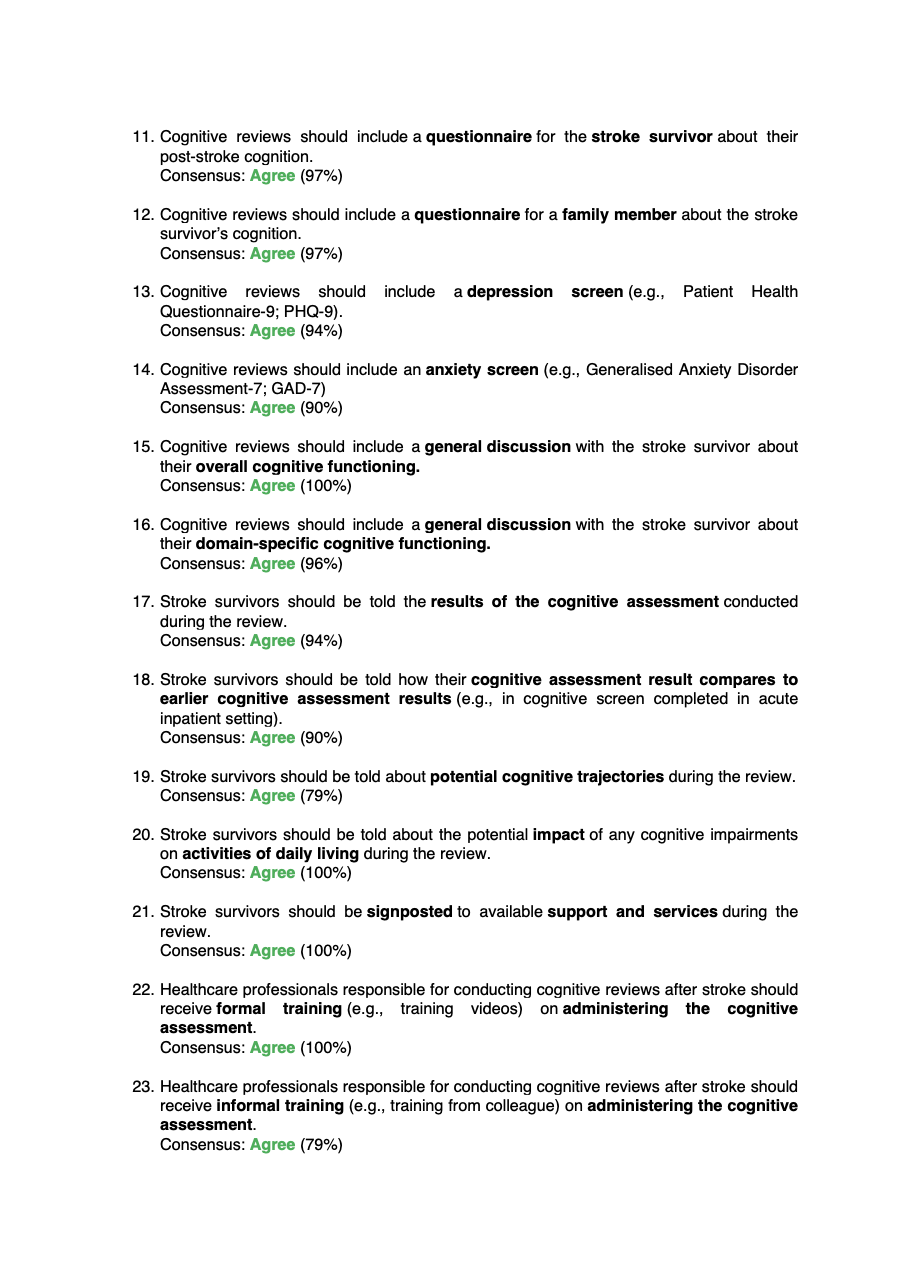
**

**
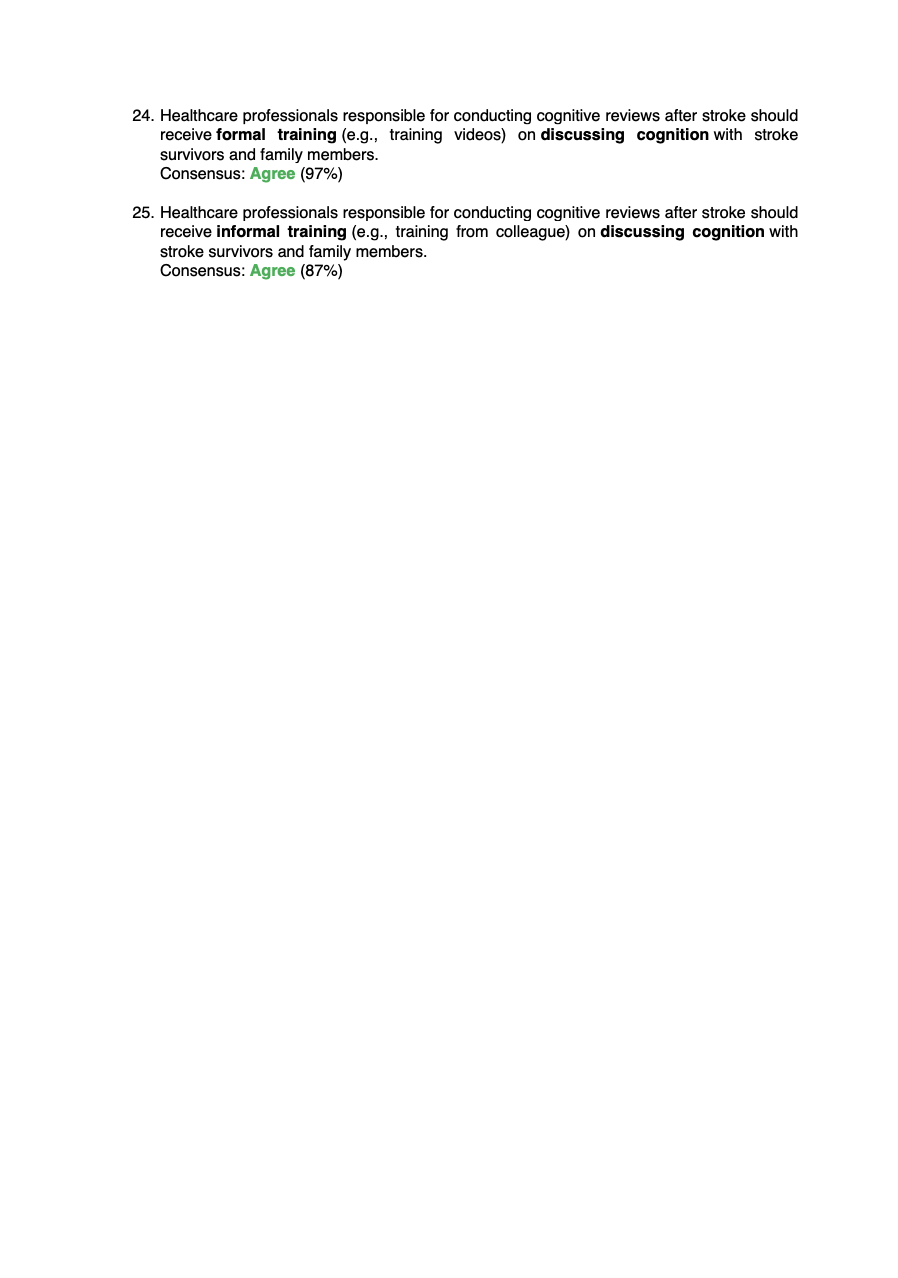
**

**Supplementary Material.** Themes developed from template analysis of free-text responses to items in the third and final modified Delphi survey round. Note that quotes are copied verbatim from participant responses with typographical errors uncorrected.

**Statement 1**

***Too late to be useful.***

A common concern among participants was that conducting a cognitive review one-year after stroke would be too late to have a meaningful impact and that it would be more beneficial for patients to receive a review at an earlier timepoint. One participant highlighted the importance of conducting a review earlier so that appropriate support can be provided, with another participant arguing that *“a lot of harm can happen in somebody's life in 12 months with unrecognised cognitive problems”* (Clinical Psychologist & Clinical Neuropsychologist).

*I feel this should be earlier within the 3-6 month mark to ensure difficulties are picked up earlier and support can be provided as needed. (Occupational Therapist)*

*Should be earlier, as by a year post someone may have lost a job or relationships due to lack of support for cognitive changes by that point. (Speech and Language Therapist)*

***Concerns about feasibility.***

Another concern was that it would not be clinically feasible to conduct follow-up reviews at one-year for all stroke survivors without cognitive impairment detected during acute hospital admission. Lack of clinical resources – including staff and time – was cited as a key challenge in implementing such reviews and one participant highlighted the *“opportunity costs”* (Physician) associated with conducting these reviews. Similarly, one participant argued that, if and when clinical resources are limited, cognitive rehabilitation should take priority over late-stage reviews for those without cognitive impairment detected acutely (Occupational Therapist).

*Screening at one year in an ideal world, but do we enough about the economic and opportunity costs associated. If someone has absolutely no cognitive issues during acute admission (unusual) and develops no new symptoms, the yield from the one year screening is likely to be low. (Physician)*

*Given the evidence of the high prevalence of dementia post stroke, I think this would be a good opportunity to follow up on this. However, the resources needed would make it impossible and I do feel resources could be better allocated on those who really need the cognitive rehabilitation. (Occupational Therapist)*

***It depends on earlier cognitive reviews.***

The survey instructions explained that “The cognitive care pathway will include at least one review of cognition a by Early Supported Discharge stroke specialist services.” Some participants argued that, if the care pathway included only one review, a review at one-year would be *“better than nothing”* (Lecturer).

*This would be too late. If only one screen is being conducted in ESD this would need to be earlier to support assessment, intervention, education and therefore return to desired ADLS.* *(Occupational Therapist)*

Several participants implied that the value of a cognitive review at one-year depended on cognitive status at earlier follow-up reviews at three- and/or six-months after stroke. Specifically, these participants felt that a one-year follow-up review would not be needed, so long as no cognitive impairment was detected during these earlier reviews.

*I think if no cognitive deficits are indicated at 3 months there is no need to review again. (Occupational Therapist)*

*Providing the patient has had rehab and there has not been any evidence of cognitive deficits and neither the patient nor the family has raised any concerns, I do not think a cognitive assessment should be done. (Occupational Therapist)*

**Statement 2**

***Downsides of remote assessments.***

Several participants highlighted the potential downsides of conducting cognitive reviews remotely, rather than in person. A frequently cited concern was that important behavioural and/or clinical information would be missed by conducting cognitive assessments remotely. Some participants worried that this could lead to either inaccurate or less informative conclusions being drawn about stroke survivors’ cognitive abilities.

*This remains a difficult question to answer as there is so much contextual information that we don't know. Interviewing someone about their cognitive abilities is certainly fine to be done by phone but I would want to see the person in order to complete any cognitive testing in order to get as much information as possible from the assessment and I think valuable clinical information is more likely to get missed remotely. (Clinical Psychologist)*

Another participant argued that building rapport is easier in person and that rapport is essential to support stroke survivors in performing optimally during any cognitive assessments.

*I still think this needs to be according to clinical need rather than patient preference - sorry! Building rapport is so much easier in person (assuming they'd not previously met the assessor) and this is important for the person to do their best during the assessment. I think it's probably also more likely that higher level difficulties would be picked up if seen in person. Not saying that remote options don't have a place, I just don't think patient preference is the right way to decide. (Doctoral Student)*

One participant expressed additional concern that, although services may start out by giving stroke survivors the choice to conduct assessments in person or remotely, the remote option may become the default, despite the downsides of this approach compared to in person testing.

*All for promoting person centred care and stroke survivors should have remote as an option. However, I fear that services may lead to remote becoming the default; and remote is not as good as in-person (Physician)*

***Importance of choice and person-centredness***

Nevertheless, some participants cited the concept of person-centred care as a reason why options should be given to conduct cognitive assessments either remotely or in person. Some participants highlighted the importance of tailoring care to individual needs, whilst acknowledging the downsides of remote testing.

*Any intervention has to be patient centred (Physician & Professor)*

*choice is important and that remote is better than nothing, but there is a risk that potentially things will be missed in remote assessments. (Occupational Therapist)*

Alongside person-centredness, the concept of informed decision-making was felt to be important. Participants suggested that healthcare professionals should offer stroke survivors the choice to receive either an in person or remote cognitive review, after clearly explaining the advantages and disadvantages of each option. In this way, it was felt that care would be person-centred but that stroke survivors would be fully informed about the costs and benefits of different approaches.

*It should be an informed choice - e.g. patients should be presented with pros and cons. (Clinical Psychologist & Lecturer)*

*I do think choice and accessibility is important therefore both videoconferencing and face to face should be offered. Not telephone consult. However the pros and cons of each should be clearly explained to the patient to enable them making an informed choice. My personal preference would be face to face but remote is better than a DNA. (Occupational Therapist)*

Several participants argued that providing choice and a person-centred approach to cognitive reviews was important to improve access to these reviews. In particular, participants felt that a remote option would improve access for stroke survivors with work and/or caring responsibilities and those living in remote areas with little access to transport.

*I am keen to ensure that access to this review is as universal as possible. For young, working age patients, or for patients living in remote areas with no access to transport, remote assessment may be the best, or indeed the only option. So, although the default option should be face to face, I strongly feel there should be other options for remote assessment (videoconference better than telephone) (Clinical Psychologist & Lecturer)*

*Stroke survivors need to be provided with options for follow-up to increase the likelihood of them engaging with this. This also avoids discriminating against those that can't get time off work or caring responsibilities. (Occupational Therapist & Doctoral Student)*

**Statement 3**

***Risk of misinterpretation in stroke populations.***

Participants who disagreed with the statement that cognitive reviews should include a dementia screen expressed concern that performance on these screens could be confounded by certain post-stroke impairments, such as neglect and aphasia.

*Standard dementia screens can potentially be misleading because of all the confounds in a stroke population i.e. neglect, dysphasia, motor problems. Whatever training you put in place, there will still be some people who take the standard cut-off (e.g. 88/100 on ACE-III) and say, 'the score is below the cut-off and this suggests the person has dementia' (Clinical Psychologist & Clinical Neuropsychologist)*

For this reason, some participants explicitly stated that a stroke-specific screen would be better.

*I don't feel it that a dementia screen is necessarily a routinely helpful element to include in the review and the results could be used inappropriately if new post-stroke changes are misinterpreted. Inclusion of a stroke-specific screen, such as the OCS, would be better. (Clinical Psychologist)*

***Preference for situation- and person-specific approach.***

Some participants agreed that a dementia screen should not be administered by default, due to the potential risk of misinterpretation, but that it could be used as an adjunct in cases where dementia was suspected to be contributing to post-stroke cognitive functioning.

*I feel that if the specialist clinician feels that dementia could be playing a role then this could be explored further, but not as default due to risk of misinterpretation etc. by others. (Clinical Psychologist)*

In line with this, some participants argued that healthcare professionals should consider the precise profile and/or longitudinal course of the person’s cognitive impairment to determine whether a dementia-type syndrome could explain it. They argued that, if the profile/course of symptoms suggested a dementia-type syndrome, administering a dementia screen could be beneficial.

*I changed my response to Disagree with a lot of reservation. I think the question is not definitive. A dementia screen would be very useful in certain situations: evidence of longitudinal cognitive decline either before stroke or months/years after the stroke; or a cognitive profile that doesn't match the stroke eg dense amnestic picture with a posterior stroke. Stroke and neurodegenerative disorders often co-exist particularly in the older patients. [...] So Dementia screen is important in some patients but not routinely in ALL patients. (Physician & Lecturer)*

One participant also suggested that healthcare professionals should consider key risk factors for dementia, implying that a dementia screen may be useful in cases where stroke survivors are at high risk for dementia.

*This depends on other factors e.g. age, risk factors for dementia etc. (Clinical Neuropsychologist)*

Making an accurate differential diagnosis between stroke-specific cognitive impairment and a dementia-type syndrome was felt to be important so that stroke survivors could be provided with appropriate rehabilitation, education, and potentially also medication, given encouraging results from recent trials of pharmacological treatments for dementia.

*If a patient has had a stroke specific cog assessment and his/her cog profile is well establish, a dementia screen could be redundant. However, for a patient with suspected or establish global cog deficits who may have had previos dementia screen, a dementia assessment a few months down the line could show a cognitive trajectory that could be useful for her/his rehab plan and family education. (Occupational Therapist)*

*It is becoming increasingly important to diagnose conditions such as Alzheimer's with the new drugs that are likely to change practice in the future. So acurate pathological diagnosis of the cognitive difficulties in these patients is going to become even more important. (Physician & Lecturer)*

**Statement 4**

***Concerns about feasibility***

A key concern among participants who disagreed with this statement was lack of clinical resources to administer neuropsychological assessments during cognitive reviews. Participants highlighted lack of both time and trained staff as key reasons why it may not be feasible to administer neuropsychological assessments during cognitive reviews.

*I don't think there will ever be enough people in stroke services to administer something that requires a more sophisticated understanding of psychometric assessment to make this workable - a 'review' will end up being too long and unwieldy. (Clinical Psychologist & Clinical Neuropsychologist)*

*I fear this would make it too long and we do not have sufficiently trained neuropsychological staff to provide it. (Occupational Therapist)*

*Given the potentially big numbers of people needing assessed, offering a neuropsych battery like this to all would need major investment - investment that is unlikely. (Physician)*

One participant suggested that, rather than conducting a neuropsychological assessment, information from other sources could be used to support a formulation.

*I would rather we do a small amount of testing and put just as much (or more) emphasis on other sources of information to support a formulation e.g. clinical interview, collateral information, functional assessment. (Clinical Psychologist & Clinical Neuropsychologist)*

***Importance of onwards referral***

Some participants acknowledged the limited clinical resources available for neuropsychological assessment, but suggested a stepped approach should be offered, whereby neuropsychological assessments are offered to stroke survivors as and when there is a clinically indicated need (e.g., according to results on a shorter screen, clinical observations, and/or self-reported problems).

*I don't think so as a standard procedure, rather a shorter screen such as the OCS should be used in the first instance. A Neuropsychological battery could be an additional step if the need is indicated by other tests or functional observations/client or relative reported problems. (Occupational Therapist)*

*A two tiered approach with initial screening/triage to select those needing more detailed assessment seems a better use of resource. (Physician)*

*I've said agree but only if clinically indicated. Not for everyone on the first appointment, it should be used if an initial screen or report indicates difficulties. It depends if you mean it should 'always include a neuropsychological assessment battery' - in which case, no but that's not to say they shouldn't ever be used. (Lecturer)*

Some participants agreed that neuropsychological assessments should be administered on a needs-led basis, but that the decision should consider personal circumstances, as well as cognitive concerns and/or performance. Whether the stroke survivor would be returning to work or education was felt to be a particularly important consideration for one participant.

*This needs to be need-led. If cognitive difficulties are being reported, or a stroke survivor is trying to return to work/college then the information from this testing can be invaluable. In other cases however it may not be needed. It should certainly be something that is available and easily accessible within the service. (Clinical Psychologist)*

*ONLY if below cut off for cognitive screen OR patient reports difficulties and is returning to cognitively demanding tasks. (Occupational Therapist)*
